# Assessment of the effectiveness and efficiency of the West Africa medicines regulatory harmonization initiative by the member countries

**DOI:** 10.3389/fphar.2022.1069345

**Published:** 2022-11-25

**Authors:** Mercy Owusu-Asante, Delese Mimi Darko, Stuart Walker, Sam Salek

**Affiliations:** ^1^ School of Life and Medical Sciences, University of Hertfordshire, Hatfield, United Kingdom; ^2^ Food and Drugs Authority, Accra, Ghana; ^3^ Centre for Innovation in Regulatory Science, London, United Kingdom; ^4^ Institute of Medicines Development, Cardiff, United Kingdom

**Keywords:** joint assessment procedure, benefits, effectiveness, efficiency, West Africa medicines regulatory harmonization project (WA-MRHA)

## Abstract

**Background:** The West Africa Health Organization launched the West Africa Medicines Regulatory Harmonization Project (WA-MRH) in 2017 with the overarching objective to improve the availability of high-quality, safe and effective medicines and vaccines by the 15 countries in the Economic Community of West African States region. Although this project has made significant progress towards the realisation of its goals, challenges still remain. The aims of this study were to evaluate the effectiveness and efficiency of the WA-MRH, examine what challenges are being encountered and identify strategies that would strengthen the process for realising the initiative’s goals.

**Methods:** The Process Effectiveness and Efficiency Rating (PEER) questionnaire was used to collect data from assessors representing the seven active NMRAs in the joint assessment procedure that identified the benefits, challenges and recommendations for improving the performance of the WA-MRH project.

**Results:** The benefits of the joint assessment procedure include time savings to manufacturers resulting from submitting one dossier and the same response package to multiple countries resulting in access to the multiple African markets within the same timeframe. Additionally, some of the NMRAs have been able to strengthen their technical capacity as a result of this initiative. Key challenges to the project include the lack of a robust information technology system that would enable dossier tracking and constraints in human resources needed to support dossier submissions and the assessment process.

**Conclusion:** This study identified the strengths of the WA-MRH initiative as well as strategies for improvement and achievement of its objectives. The centralised submission of a dossier and its tracking is key to the regulatory assessment process. This research has demonstrated that amongst other considerations, a robust information technology system, coupled with the necessary human resource capacity would greatly enhance the effectiveness and efficiency of the WA-MRH initiative.

## 1 Introduction

The national medicines regulatory authorities (NMRAs) in Africa are challenged to judiciously utilise their limited human, technical and financial resources to ensure access to safe, high-quality and efficacious medicines in the presence of high disease burden and inadequate local pharmaceutical manufacturing on the continent (World Health Organization, 2010; World Health Organization, 2014). To help address these challenges in Africa, the African Medicines Regulatory Harmonization (AMRH) Initiative was launched in 2009 to collaborate with the Regional Economic Communities to establish mechanisms to harmonize regulatory activities in the various regional blocks. Subsequently, in 2010, a report by 26 NMRAs in sub-Saharan Africa, which had been assessed over an 8-year period was published by the World Health Organization (WHO, 2010). Not surprisingly, the common challenge that was reported was inadequate regulatory capacity. To deal with this challenge, the West African Health Organization and its economic partners took a bold decision in 2014 to initiate medicine regulatory harmonisation in West Africa under the leadership of WAHO. As part of preparations for the commencement of the West African harmonisation programme, a Steering Committee, made up of the heads of medicine regulatory authorities in the 15 countries in West Africa, was established in 2015 to provide the much-needed high-level regulatory support required for the initiative to be rolled out successfully. Following this, in November 2017 the West African Health Organization (WAHO), launched the West Africa Medicines Regulatory Harmonization Project (WA-MRH) under the AMRH, to improve the availability of high-quality, safe and effective medicines and vaccines in the Economic Community of West African States (ECOWAS) (West Africa Medicines Regulatory Harmonization, 2021). According to the Director General of the West African Health Organization (WAHO), “that is why we have agreed to jointly register and regulate medicines produced locally and imported into the region with the aim of reducing the time of registration and improving access to medicines as well as ensure better regulatory oversight.” (Daniel, 2019). This remark referred to the challenges with technical and financial resources and also differences in the official national languages in the ECOWAS region (Daniel, 2019).

Between March 2018 and February 2019, harmonised guidance documents which were required to facilitate the initiative were developed by technical working groups, and then authorised by the Steering Committee (West Africa Medicines Regulatory Harmonization, 2021).

In the current operating model of the WA-MRH initiative, an NMRA serves as a lead coordinator for a 2-year period and receives, validates and arranges for the assessment of the dossiers and additionally communicates with applicants and the WA-MRH secretariat, which is based in the WAHO. There are 11 steps in the WA-MRH joint assessment procedure which include: expressions of interest; pre-submission meeting; submission and dossier validation; technical evaluation (Phase I); joint evaluation by the expert working group (EWG) and technical partners (Phase I); joint good manufacturing practice inspection and quality control; technical evaluation (Phase II); joint evaluation by expert working group and technical partners (Phase II); technical evaluation (Phase III); final joint evaluation by EWG and technical partners; and validation by WA-MRH Steering Committee (WA-MRH, 2021).

**FIGURE 1:**
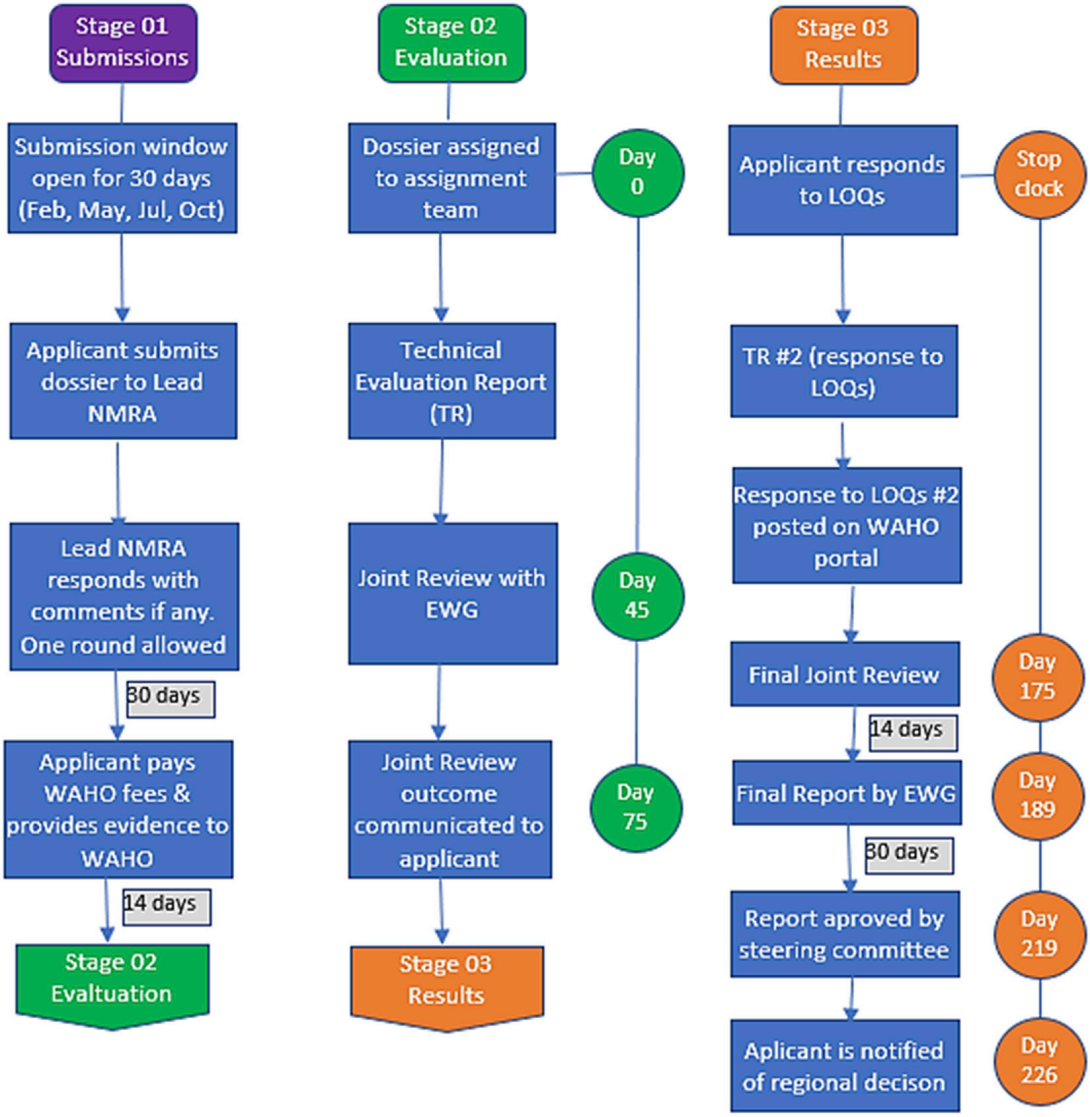
The WA-MRH joint assessment process.

A flow chart of the WA-MRH joint assessment procedure is provided (Figure 1). In summary, it takes 120 and 226 calendar days for a high standard-completed dossier and a dossier with a one-time list of questions to go through these 11 steps, respectively (WA-MRH, 2021).

Since 2019, seven NMRAs in West Africa have participated in joint assessments of submitted applications for registration of medicines and the outcomes of these assessments have been taken as a basis for the regulatory decisions in the 15 NMRAs in the ECOWAS region. It is important to note that in the ECOWAS region, the NMRAs of Ghana and Nigeria obtained WHO-GBT maturity level-3 status in April 2020 and April 2022 respectively, a level that indicates a stable and well-functioning regulatory system (WA-MRH, 2021; WHO, 2022a).

There is a drive within regulatory agencies to re-engineer their processes to meet stakeholders’ expectations in a timely manner. This timeliness, being central to assessing the efficiency and effectiveness of any system, can be regarded as the motivation for the regular evaluation of the processes, which is to ensure that the strengths of the system are sharpened whilst identified redundancies are eliminated to realise stakeholder expectations.

Following the successful assessment of the ZaZiBoNa and EAC-MRH initiatives in 2021 and the impending launch of the African Medicines Agency, it is timely that the WA-MRH initiative is assessed at this time and hence the implementation of this study (Sithole et al., 2022; Ngum et al., 2022). The study aimed to assess the effectiveness and efficiency of the West Africa Medicines Regulatory Harmonization Initiative by the member countries. In addition, the study objectives were: obtain the views of the individual medicines’ regulatory authorities of the WA-MRH initiative about the performance of the programme to date; identify the challenges experienced by individual authorities throughout the life cycle of the WA-MRH initiative; determine the strengths and weaknesses of the initiative; identify the ways of improving the performance of the work-sharing programme; and envisage the strategy for moving forward.

## 2 Materials and methods

### 2.1 Study participants

All seven active NMRAs of the WA-MRH initiative namely, National Pharmaceutical Regulatory Agency-Burkina Faso, Ministry of Public Health- Republic of Cote d’Ivoire, Food and Drugs Authority (Ghana-FDA), National Agency for Food and Drug Administration and Control (NAFDAC) -The Federal Republic of Nigeria, Ministry of Health and Social Welfare-Republic of Senegal, Pharmacy Board of Sierra Leone (PBSL) and the Directorate of Pharmacy, Medicine and Laboratories - Togo, participated in the study between January and June 2022.

### 2.2 Data collection

The Process Effectiveness and Efficiency Rating (PEER) Questionnaire, previously developed and validated by Ngum and others to evaluate the performance of the East African Community joint assessment procedure (Ngum et al., 2022), was used to collect the study data. The PEER Questionnaire consists of five categories: 1) Authority resources; 2) Benefits of the WA-MRH initiative; 3) Challenges of the WA-MRH initiative; 4) Improving the performance (effectiveness and efficiency) of the work-sharing programme; and 5) Strategy for moving forward.

The focal person and the head of the NMRA from each country were responsible for completing and approving each questionnaire respectively. Semi-structured interviews using a checklist were carried out with each authority to validate their responses to the questionnaire. The interviews provided flexibility and a further opportunity for the respondents as they were able to give open-ended answers to some questions. Some sections of the questionnaire were clarified, challenges in completing the questionnaire were discussed, the benefits of the study acknowledged and the participants reviewed the final study report. To ensure confidentiality, the questionnaire was marked as confidential and this was reinforced during the interviews.

## 3 Results

For the purpose of clarity, the results are presented in five parts: 1) Demographics and administrative resources; 2) Benefits of the WA-MRH initiative; 3) Challenges of the WA-MRH initiative; 4) Improving the performance of the work-sharing initiative; and 5) Strategies for moving forward.

### 3.1 Part 1. Demographics, technical and administrative resources

The age of the respondents ranged from 42 to 50 years and two of the seven respondents were female. The number of years of regulatory experience ranged from 7 to 21 years Table 1 summarises the technical and administrative resources available in each of the participating NMRAs.

**TABLE 1:** Technical and administrative resources of NMRAs.

	Countries						
	Burkina Faso	Cote d’Ivoire	Ghana	Nigeria	Senegal	Sierra Leone	Togo
**Number of assessors**	27	14	32	12	30	8	30
**Number of assessors involved in WA-MRH**	8	2	5	5	5	1	2
**Keeps separate record of WA-MRH applications**	No	No	Yes	Yes	Yes	Yes	No

### 3.2 Part 2. Benefits of the WA-MRH initiative

The benefits of the initiative identified by the NMRAs were the harmonisation of registration requirements across the region, information sharing among regulators and building of capacity for assessments. Leadership commitment and governance structure was selected by half of the respondents as being beneficial, while shorter timelines for approval and clear operating model were also selected by some of the respondents. It is important to note that the benefit of harmonisation of registration requirements in the region was echoed by all the respondents.

#### 3.2.1 Strengths of the WA-MRH process for recommending the registration of products

The respondents stated that the strengths of the WA-MRH process for recommending the registration of products included regular committee meetings enabling timely finalisation of products after WA-MRH recommendation, resource savings in time and funding, priority review of WA-MRH products, as well as having a pool of expert reviewers. According to the WA-MRH process, four quarterly joint assessment meetings are held in each year. Applicants must respond to queries arising from the assessment meeting within 60 days after the first joint assessment and 30 days after the second joint assessment. It worth noting that expert reviewers are included from the NMRAs in Ghana and Nigeria; both having achieved WHO-GBT maturity level- three status and therefore the process can be considered to be adequately resourced regarding regulatory capacity.

#### 3.2.2 Benefits of the WA-MRH initiative to NMRAs

**FIGURE 2:**
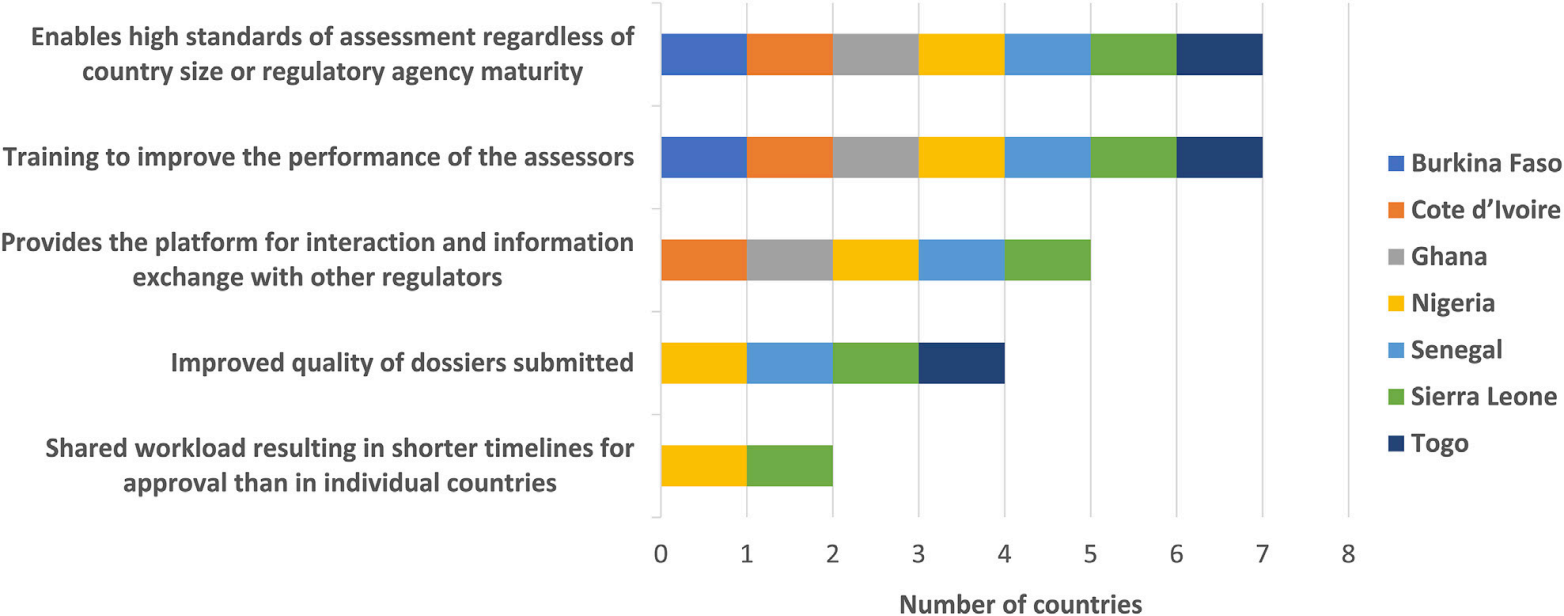
WA-MRH benefits to member countries (regulators).

The NMRAs reported that the WA-MRH work-sharing initiative has enabled applications of high standards of assessment regardless of size of country or maturity of regulatory agency. The training to improve the performance of the assessors provided the platform for interaction and information exchange with other regulators. The improved quality of dossiers submitted as well as a shared workload resulted in shorter timelines for approval than in individual countries (Figure 2). The results showed that the NMRAs had identified all the benefits of this initiative.

#### 3.2.3 Benefits of the WA-MRH initiative to applicants

**FIGURE 3:**
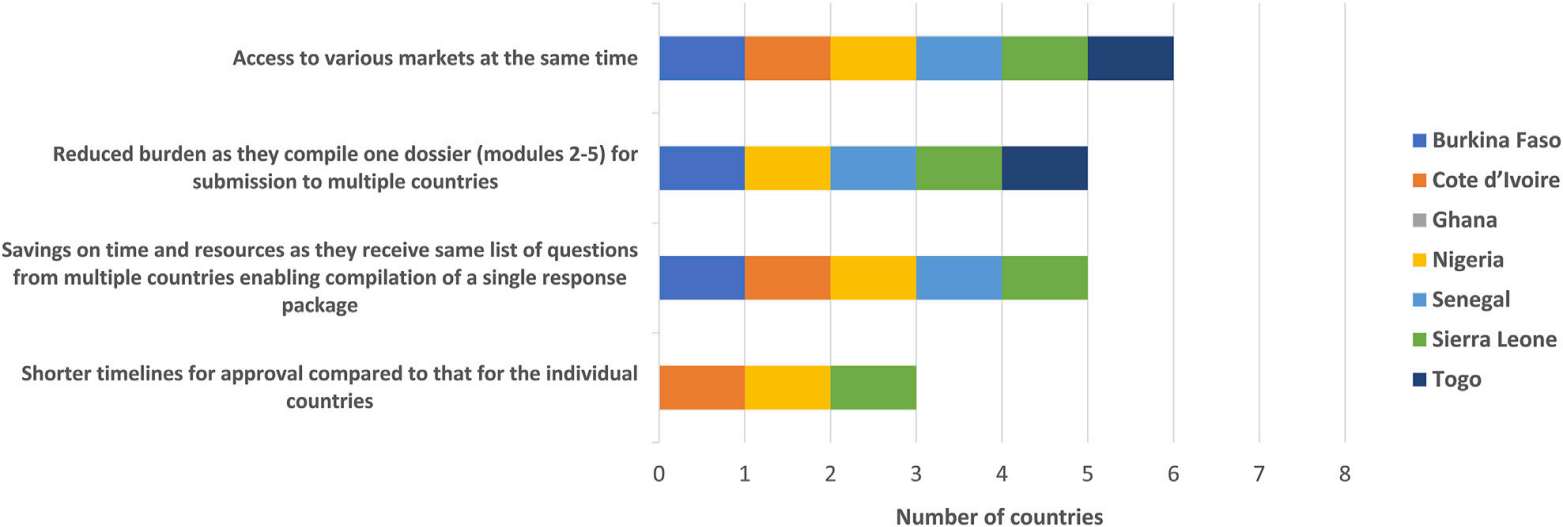
WA-MRH benefits to manufacturers (applicants).

The benefits of the WA-MRH initiative for applicants included access to various ECOWAS markets at the same time, a reduced burden as they compile one dossier (modules 2–5) for submission to multiple countries, the savings in time and resources as they receive the same list of questions from multiple countries, enabling the compilation of a single response package as well as shorter timelines for approval compared with that for the individual countries (Figure 3).

#### 3.2.4 Benefits of the WA-MRH initiative to patients at the country or regional level

The NMRAs reported quicker access to quality assured medicines and increased availability of medicines as the benefits of the WA-MRH work-sharing initiative for patients at either the country or regional level. These two benefits give a good indication that the WA-MRH initiative is moving in the right direction.

### 3.3 Part 3. Challenges of the WA-MRH initiative

**FIGURE 4:**
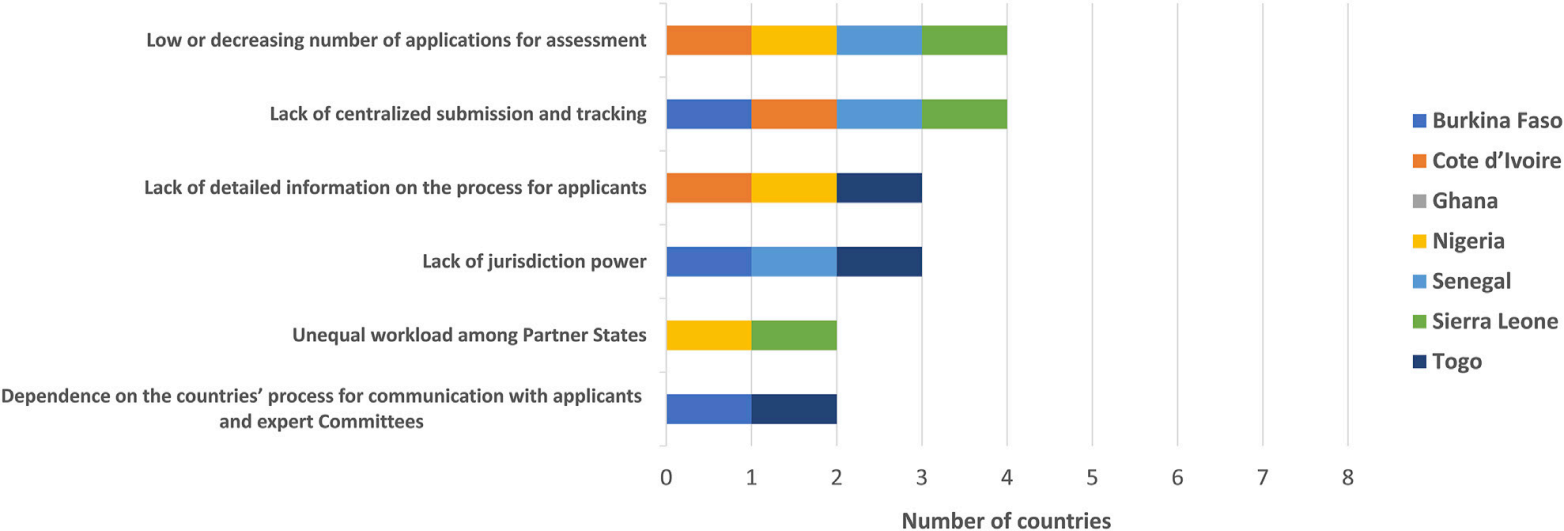
Challenges of the WA-MRH initiative.

The challenges the WA-MRH initiative identified by the NMRAs included the low or decreasing number of applications for assessment, a lack of centralised submission and tracking, a lack of detailed information on the process for applicants, a lack of jurisdiction power, unequal workload among the agencies and the dependence on the countries’ process for communication with applicants and Expert Committees. Poor IT infrastructure to support dossier submissions and the assessment process was also presented as another challenge of the WA-MRH initiative (Figure 4). The results have highlighted the challenges faced by the NMRAs at this time.

#### 3.3.1 Challenges faced at the country level in assessing/finalising WA-MRH products

**FIGURE 5:**
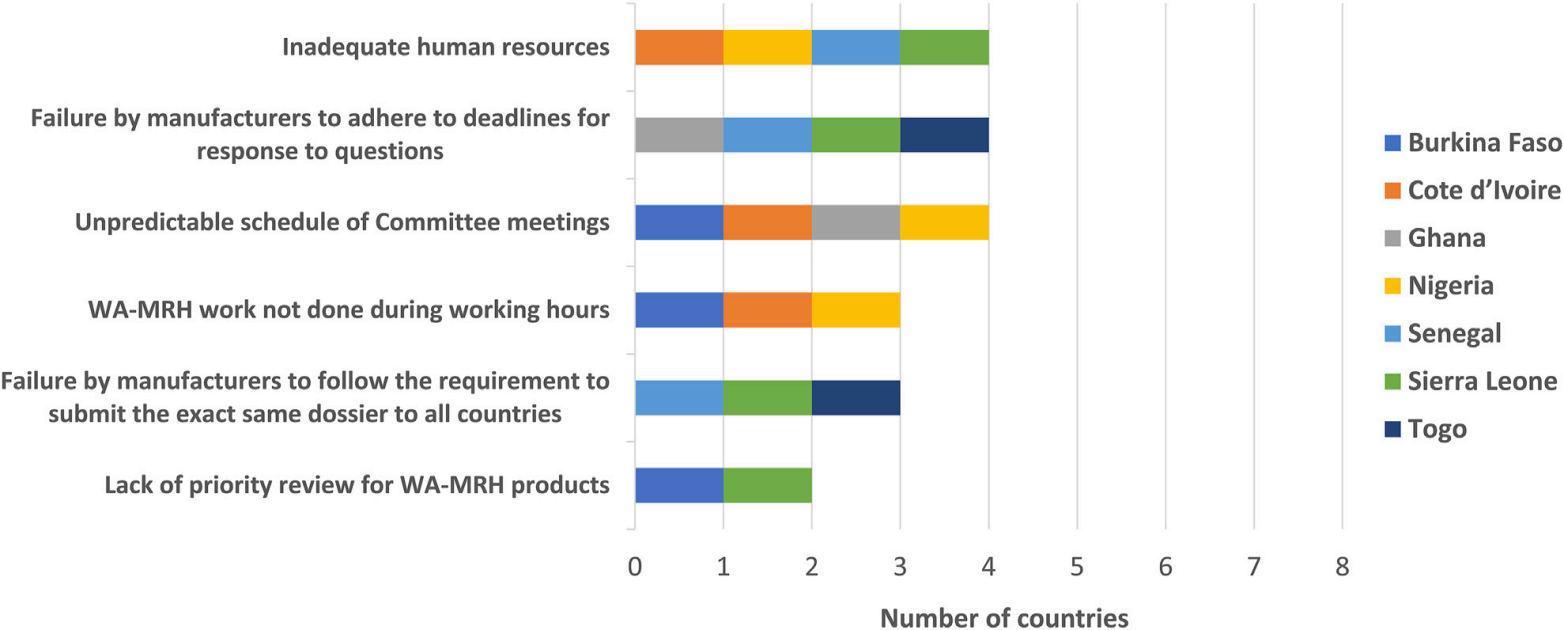
Challenges faced at country level in assessing/finalising WA-MRH products.

The views of the respondents regarding the challenges faced at the country level in assessing/finalising WA-MRH products included inadequate human resources, a failure by manufacturers to adhere to deadlines for response to questions, the unpredictable schedule of Committee meetings, the WA-MRH initiative not being recognised as part of the agency work to be carried out during working hours, the failure by manufacturers to follow the requirement to submit the exact same dossier to all countries of interest and a lack of priority review for WA-MRH products. In addition, other challenges faced at the country level in assessing/finalising WA-MRH products included the lack of a WA-MRH calendar of activities to help avoid conflicts and the lack of compatibility of the time limits for the joint assessment procedure with the national procedures (Figure 5).

#### 3.3.2 Challenges faced by applicants submitting applications to the WA-MRH initiative

The challenges faced by the applicants identified by the NMRAs, were that the WA-MRH process is more stringent than some country processes, the differing labelling requirements in participating countries, the lack of clarity about the process for submission and follow-up in each country as well as the lack of information on country websites and the WA-MRH website about the process, milestones and timelines, as well as pending and approved products. The results suggest that the applicants have shared the type of challenges faced by them with the respective NMRAs.

### 3.4 Part 4. Improving the performance (effectiveness and efficiency) of the work-sharing programme

#### 3.4.1 Ways to improve the effectiveness of the WA-MRH initiative

**FIGURE 6:**
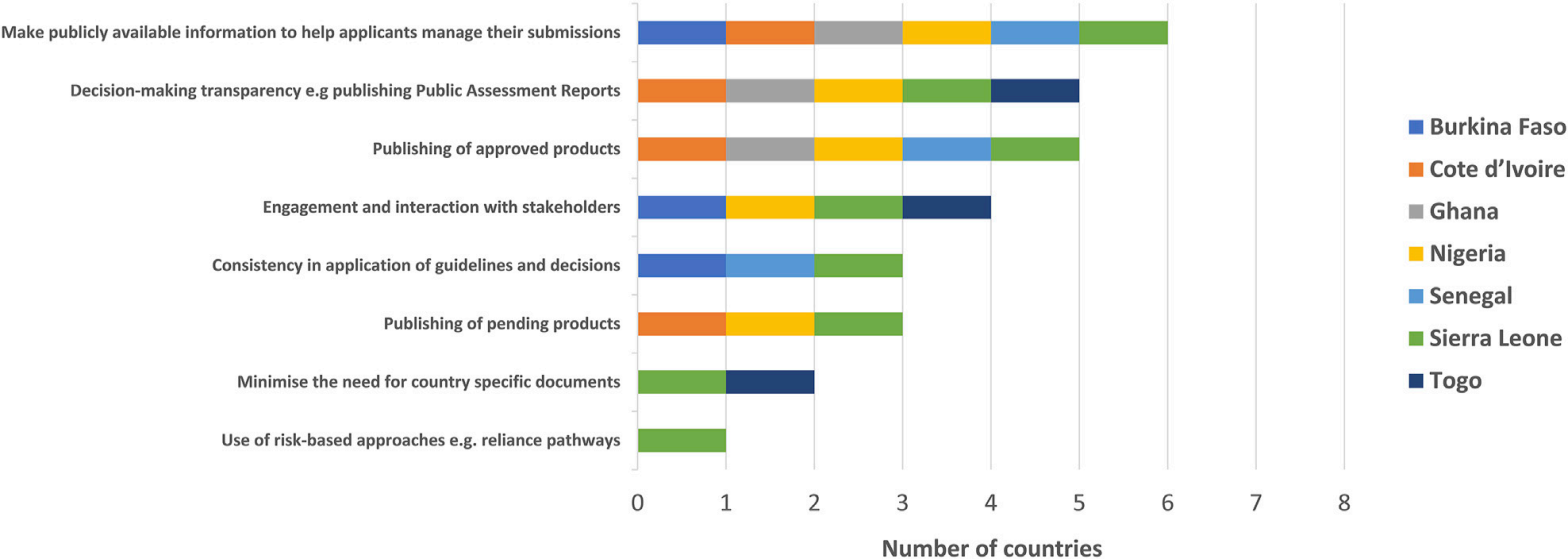
Ways to improve effectiveness of the WA-MRH initiative.

The NMRAs acknowledged that there are multiple options to be considered in order to improve the effectiveness of the WA-MRH initiative. These included making any information publicly available that might help applicants in managing their submissions, such as templates of documents, lists of questions and answers, timelines and milestones, disclosure of internal SOPs, decision-making transparency such as publishing Public Assessment Reports, publishing of lists of approved products, engagement and interaction with stakeholders, consistency in application of guidelines and decisions, publishing of pending products, minimising the need for country-specific documents and the use of risk-based approaches such as reliance pathways (Figure 6).

#### 3.4.2 Ways to improve the efficiency of the WA-MRH initiative

**FIGURE 7:**
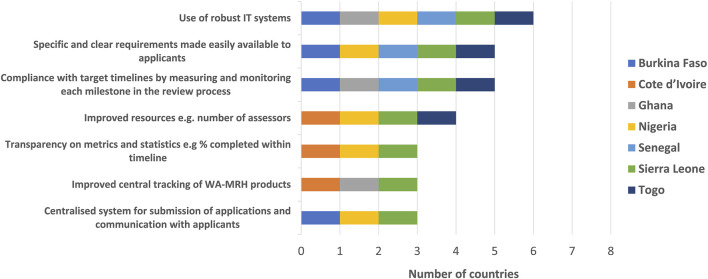
Ways to improve efficiency of the WA-MRH initiative.

Ways to improve the efficiency of the WA-MRH initiative were suggested by the NMRAs, which included the use of robust IT systems, specific and clear requirements made easily available to applicants, compliance with target timelines by measuring and monitoring each milestone in the review process, improved resources; for example, number of assessors, transparency on metrics and statistics; for example, percentage of reviews completed within prescribed timelines, improved central tracking of WA-MRH products and a centralised system for submission of applications and communication with applicants. Expanding the Expert Committees to include more resources available in the region was also presented as an additional way to improve the efficiency of the WA-MRH initiative (Figure 7).

### 3.5 Part 5. Strategy for moving forward

Finally, possible strategies that were considered most effective in improving efficiency were to continue with the current operating model but provide full information on the process including timelines and milestones as well as approved products on every participating country’s website as well as on the WA-MRH website and the establishment of a regional administrative body to centrally receive and track WA-MRH applications, which would be responsible for allocating work, apportioning the applicable fees to countries, tracking of applications and communication with applicants. The following suggestions were made by a respondent;

“We need to establish communication channels with regulatory agencies in the EU, US as well as the WHO to facilitate reliance-based registrations. This will help cut down on the time expended in reviews especially for the active pharmaceutical ingredient which may have been previously accepted in these regions”

“Also, in the medium to long term, there is a need to encourage the inclusion of regulatory sciences in higher institutions in the ECOWAS region. There is still a significant gap in knowledge as it concerns regulatory requirements amongst most manufacturers in the ECOWAS region and this is evidenced by poorly organized product dossiers submitted for registration in most countries.”

## 4 Discussion

The WA-MRH initiative has been of value, with the most outstanding benefit being the harmonisation of registration requirements within the sub-region. This is of great value to both NMRAs and manufacturers, as it allows the standardisation of the criteria for submission of applications by manufacturers and the assessment by the NMRAs. Whilst the “enthusiasm and commitment of ECOWAS, NMRAs and the pharmaceutical industry toward the implementation of a harmonized medicine regulatory system” for the sub-region have been noted (Kamwanja et al., 2011), it has also been observed that similarly to status of the ZaZiBoNa initiative, the important benefit of shorter timelines for approval has not been achieved at this time. A solution for this shortcoming should therefore be given a high priority. Lessons can be taken from the EAC-MRH initiative which has achieved the important benefit of shorter timelines for approval since this was not an outcome for the ZaZiBoNa initiative (Sithole et al., 2022; Ngum et al., 2022). This will enhance the reported benefit of the WA-MRH initiative to patients at the country and regional levels of having quicker access to quality-assured medicines.

It is of interest to note that each of the NMRAs involved in the joint assessment procedure make from 7% to 42% of assessors available to support the WA-MRH initiative, with assessors from Ghana and Nigeria contributing 36% of the total pool of assessors for this initiative. Since some countries are not adequately resourced to be able to contribute their requisite share of assessors to support this initiative, it is appropriate that the relatively better-resourced NMRAs continue to make available more of their assessors for the initiative. It is hoped that as other NMRAs are strengthened, this will result in a positive effect in the WA-MRH initiative, including shorter timelines.

Data available at the end of this study (June 2022) showed that the review and decision for seven applications to the WA-MRH initiative have been completed. Being the most recently implemented joint assessment procedure on the African continent, the WA-MRH initiative is in its early days in comparison to the ZaZiBoNa and EAC-MRH initiatives. Since only a few applications have been finalised and there is a decreasing number of applications for assessment, a further study should be conducted, possibly by engaging the manufacturers to learn about their challenges and encourage their active participation so that more medicines become readily available to patients in the ECOWAS region through the initiative. Valuable lessons and experiences can be drawn from the WHO Prequalification of medicines programme, which has been remarkably successful with expanding its portfolio to reach other unmet needs in an effort to cover a wide range of medicines required for public health (World Health Organization, 2022b).

It is important to note that other challenges of the WA-MRH initiative such as lack of centralised submission and tracking and a poor IT infrastructure to support dossier submissions and the assessment process can be considered as common with the other MRH initiatives in Africa as these challenges were also reported by Sithole and others and also by Ngum and others (Sithole et al., 2022; Ngum et al., 2022). In addition to providing a robust IT infrastructure to track dossier assessments, the competence of assessors should be adequate to perform to international standards and the fast-tracking of applications should be entertained only when public health rather than the manufacturers’ wishes requires such prioritisation (Hill and Johnson, 2004).

For the WA-MRH to be successful, other mechanisms should be considered, such as making any information that might help applicants in managing their submissions publicly available (templates of documents and lists of questions and answers), and providing decision-making transparency through such means as publishing Public Assessment Reports as well as lists of approved products. The need for these mechanisms, which were also reported by Sithole and others and again by Ngum and others (Sithole et al., 2022; Ngum et al., 2022) confirm the similarity of issues associated with these initiatives being implemented across the different subregions in Africa. A study of the challenges affecting some of the harmonisation initiatives being implemented in other parts of the world would also be of value (Mendez and Trejo, 2020).

It is timely to note that medicine harmonisation initiatives and effective collaborative mechanisms amongst NMRAs can promote efficient utilisation of limited human, technical and financial resources to perform regulatory activities to improve patients’ access to medicines in West Africa as well as other parts of the continent (Azatyan, 2013; African Union Development Agency-New Partnership for Africa’s Development, 2022; Mukanga, 2018).

Finally, the majority of the NMRAs regarded the establishment of a regional administrative body, if legally possible, as the best strategy to improve performance going forward. Some of the reasons they have suggested to support this would include: Promotion of mutual recognition of decisions by other NMRAs which would also reduce the time limit for granting marketing authorisations; having staff dedicated exclusively to the agency; relieving some regulatory burden from participating countries; if properly established, without conflicts with national sovereignty, the ECOWAS regional medicines agency would improve the quality of medicines available in the region, and also facilitate the centralised registration of products to improve access to medicines and help coordinate pharmacovigilance activities and control substandard products in the region; promote culture of accountability and transparency; and making it possible to save material, technical and financial resources, preventing bottlenecks in the approval process at the NMRA level. However, it will also be necessary to maintain operational and efficient NMRAs to guarantee the quality, safety and effectiveness of the medicines that do not fall within the framework of the centralised procedure.

There was, however, a suggestion by the NMRAs that the current system should be strengthened first, since the creation of a regional agency may not be required in view of the decision to establish an African Medicines Agency.

The authors’ key recommendations to strengthen the WA-MRH initiative going forward are: **Digitalization of regulatory processes** - Availability of a robust IT system would facilitate a centralised system for submission of applications and communication with applicants; **Promotion of regulatory reliance mechanisms -** These mechanisms will reduce or eliminate duplication in dossier assessments and ultimately lead to shorter approval timelines at the regional level; **Bridging the gap in academia by providing current knowledge in regulatory science -** The academic institution should be encouraged to provide relevant and current courses to support pharmaceutical regulations in the region; and **Training of more assessors to increase human resource capacity in the region, especially in lesser-matured regulatory authorities** - This would go a long way to positively impact the effectiveness and efficiency of this initiative.

The scope of this study was limited to the process and operating model of the WA-MRH initiative. In addition, there were only seven applications assessed by the initiative during the 3 years of its operation and a small number of the member countries were involved in such assessment. However, this early evaluation of the effectiveness and efficiency of the initiative is instrumental in identifying the achievements and the challenges moving forward, as more of the seven member countries become engaged in the assessment of applications. Going forward, it would be helpful to obtain quantitative data to support these views. Such data would include actual metrics of the time taken to register the medicines in NMRAs following a recommendation from the WA-MRH initiative.

## 5 Conclusion

This study identified the benefits and challenges of the WA-MRH initiative as experienced by the NMRAs as well as the options available to improve its effectiveness and efficiency. The key recommendations which have been proposed, if implemented, should further strengthen this initiative to enable it to fulfil its core mandate which is to “Improve the availability of quality, safe and effective medicines and vaccines in the ECOWAS region”.

## Data Availability

The raw data supporting the conclusions of this article will be made available by the authors, without undue reservation.

## Abbreviations

EAC-MRH, East African Community- Medicines Regulatory Harmonization; ECOWAS, Economic Community of West African States; NMRAs, National Medicines Regulatory Authorities; PEER, Process, Effectiveness and Efficiency Rating; WAHO, West African Health Organization; WA-MRH, West Africa Medicines Regulatory Harmonization; WHO-GBT, World Health Organization Global Benchmarking Tool; ZaZiBoNa, Zambia Zimbabwe Botswana Namibia.
