# Supplementary material for: Assessment of the effectiveness and efficiency of the West Africa medicines regulatory harmonization initiative by the member countries
Source: Front Pharmacol. 2022 Nov 25;13:1069345. doi: 10.3389/fphar.2022.1069345 (PMC9732020; doi:10.3389/fphar.2022.1069345)
Supplement: Supplementary file 1 [file DataSheet1.PDF]

CONFIDENTIAL

WA-MRH JOINT ASSESSMENT PROCEDURE

PROCESS EFFECTIVENESS & EFFICIENCY RATING (PEER)

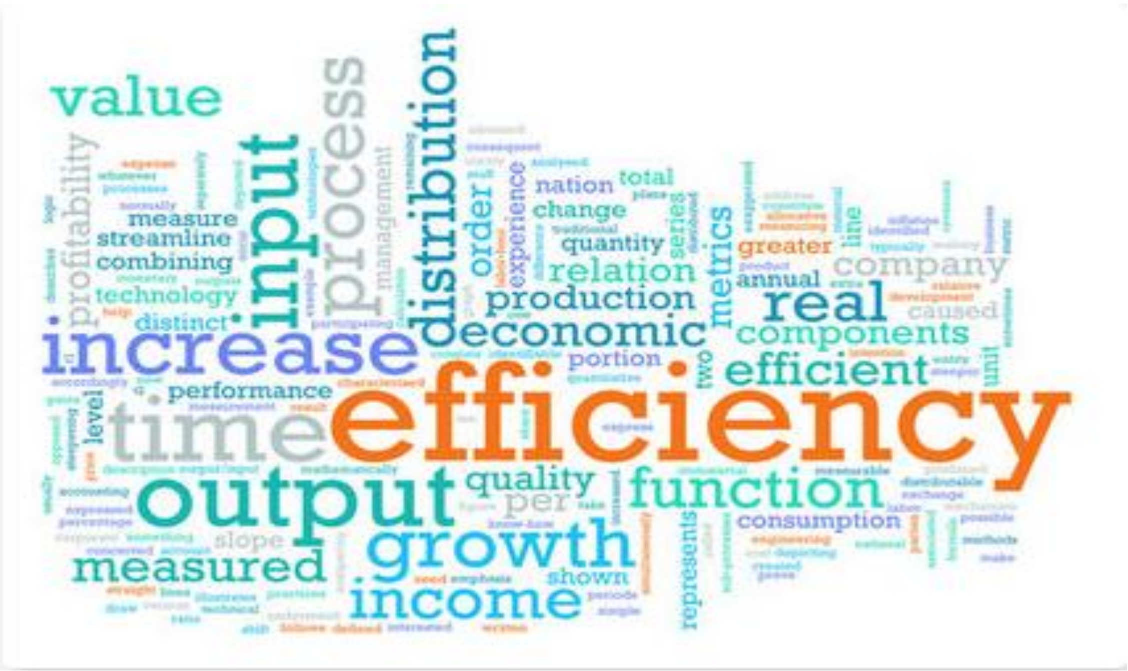

Pic taken from https://www.referenceforbusiness.com/management/De-Ele/Effectiveness-and-Efficiency.html

PEER QUESTIONNAIRE

November 2021

Contacts:

Mercy Owusu-Asante
mercy.owusu-asante@fda.gov.gh

Prof Stuart Walker
swalker@cirs.org

Prof Sam Salek
sssalek52@gmail.com

INTRODUCTION

The launch of the West Africa Medicines Regulatory Harmonization Project (WA-MRH) in July 2017 was to improve the availability of quality, safe and effective medicines and vaccines in the ECOWAS region.

At least seven (7) National Medicines Regulatory Agencies (NMRAs) have participated in joint assessments of submitted applications for registration of medicines and taken the outcome as a basis for the regulatory decisions in the 15 NMRAs in the ECOWAS region.

In recent years, there has been a drive within regulatory agencies to re-engineer their processes for improved efficiency and effectiveness and this often begins with a baseline evaluation of the current process to identify strengths and weaknesses. *Effectiveness* can be defined as 'doing the right things' often measured by the value derived by customers/stakeholders from an organisation's processes or services while *Efficiency* can be defined as 'doing things right' which saves the organization time and resources.

Study Participants

The PEER Questionnaire is being sent to 7 National Medicines Regulatory Authorities in the ECOWAS region namely, Pharmacy Board of Sierra Leone (PBSL), National Pharmaceutical Regulatory Agency-Burkina Faso, Ministry of Public Health- Republic of Cote d'Ivoire, Ministry of Health and Social Welfare-Republic of Senegal, National Agency for Food and Drug Administration and Control (NAFDAC)-The Federal Republic of Nigeria, Food and Drugs Authority (Ghana-FDA) and the Directorate of Pharmacy, Medicine and Laboratories- Togo

AIM

The aim of this study is to evaluate the effectiveness and efficiency of the current operating model of the WA-MRH initiative including the challenges it faces as well as identifying opportunities for improvement.

STUDY OBJECTIVES

1. Obtaining the views of the individual medicines' regulatory authorities of the WA-MRH initiative about the performance of the programme to date.
2. Identifying the challenges experienced by individual authorities throughout the life cycle of the WA-MRH initiative.
3. Determining the strengths and weaknesses of the initiative
4. Identifying the ways of improving the performance of the work sharing programme.
5. Envisaging the strategy for moving forward

CONFIDENTIALITY

Thank you for agreeing to participate in this survey. **Your responses will be treated in strictest confidence and no identifiers of countries or respondents will be shared with any third party or made public**. External reports or presentations of the data will be kept confidential.

The questionnaire is divided into five short sections and will take 20 minutes to complete. Thank you for taking time to complete it. We value your input!

A. DEMOGRAPHICS

1. Please state the name of your country ________________________
2. Please provide your responses to the following questions by writing your answer in the space provided or ticking the relevant box.
   a. Age: ________years
   b. Sex: ☐ Male ☐ Female
   c. Number of years of regulatory experience: _______years
3. What is the total number of staff in your agency? _______
4. What is the number of reviewers of marketing authorization applications? _______
5. How many reviewers participate in the WA-MRH joint assessments? _____
   ____
6. Does your agency have a separate record of applications received for assessment under WA-MRH? ☐ Yes ☐ No

B. VIEWS ON THE BENEFITS OF THE WA-MRH INITIATIVE

Select your answers by ticking the relevant box(es)

1. In your view, what are 3 (or more) benefits of the WA-MRH initiative to date?

   ☐ Leadership commitment/Governance structure
   ☐ Clear Operating Model
   ☐ Shorter timelines for approval
   ☐ Information sharing among regulators

☐ Building of capacity for assessments

☐ Sustainable resource base because of self-funding by countries

☐ `Harmonisation of registration requirements across the region

☐ Other (Please specify) ______________________________________

2. What would you say are 3 (or more) <u>strengths of your WA-MRH process</u> for recommending the registration of products?

☐ Separate register and tracking of WA-MRH products

☐ Priority review of WA-MRH products

☐ Information on the submission process and timelines for WA-MRH products are available on your country website

☐ Products approved under WA-MRH are available on your country website

☐ Regular Committee meetings enabling timely finalisation of products after WA-MRH recommendation

☐ Resource savings (time and funding)

☐ Pool of expert reviewers

☐ Other (Please specify) ______________________________________

3. How has the WA-MRH initiative benefited <u>member countries (regulators)</u>?

☐ Training to improve the performance of the assessors

☐ Provides the platform for interaction and information exchange with other regulators

☐ Shared workload resulting in shorter timelines for approval than in individual countries

☐ Enables application of high standards of assessment regardless of size of country or maturity of regulatory agency

☐ Improved quality of dossiers submitted

☐ Other (Please specify) ______________________________________

4. How has the WA-MRH initiative benefited <u>manufacturers (applicants)</u>?

☐ Reduced burden as they compile one dossier (modules 2 -5) for submission to multiple countries

☐ Savings on time and resources as they receive same list of questions from multiple countries enabling compilation of a single response package

☐ Shorter timelines for approval compared to that for the individual countries

☐ Access to various markets at the same time

☐ Other (Please specify) ____

5. How has the WA-MRH initiative benefited patients in your country or in the ECOWAS region?

☐ Quicker access to quality assured medicines

☐ Reduced prices of medicines

☐ Increased availability of medicines

☐ Other (Please specify) ____

C. VIEWS ON CHALLENGES OF THE WA-MRH INITIATIVE

Select your answers by ticking the relevant box(es)

1. In your view, what are 3 (or more) challenges of the WA-MRH initiative?

☐ Lack of detailed information on the process for applicants

☐ Low or decreasing number of applications for assessment

☐ Unequal workload among Partner States

☐ Dependence on the countries' process for communication with applicants and expert Committees

☐ Lack of centralised submission and tracking

☐ Lack of jurisdiction power

☐ Other (please specify) _Poor IT infrastructure to support dossier submissions and the assessment process_________________________

2. In your view, what are 3 (or more) challenges that you face at country level in assessing/finalising WA-MRH products?

☐ Inadequate human resources

☐ Poor record keeping and tracking of WA-MRH products

☐ Lack of priority review for WA-MRH products

☐ WA-MRH work not recognized as part of agency work to be done during working hours

☐ Unpredictable schedule of Committee meetings

☐ Lack of buy-in from expert Committee(s)

☐ Failure by manufacturers to follow the requirement to submit the exact same dossier to all countries of interest

☐ Failure by manufacturers to adhere to deadlines for response to questions

☐ Other (Please specify) Lack of a calendar of WA-MRH activities that factors in NRA activities with an aim to avoid any conflicts

3. What are the challenges faced by manufacturers submitting applications to the WA-MRH initiative?

☐ Differences in time to implementation of WA-MRH recommendations by Partner States.

☐ Lack of clarity about the process for submission and follow up in each Partner State

☐ Lack of information on country websites and the WA-MRH website about the process, milestones, timelines, pending and approved products

☐ WA-MRH process is more stringent than some country processes

☐ Differing labeling requirements in participating countries

☐ Other (Please specify) ______________________________________

D. IMPROVING THE PERFORMANCE (EFFECTIVENESS AND EFFICIENCY) OF THE WORK SHARING PROGRAMME

Select your answers by ticking the relevant box(es)

Effectiveness can be defined as 'doing the right thing' often measured by the value derived by customers/stakeholders from an organisation's processes or services while *Efficiency* can be defined as 'doing things right' which saves the organization time and resources.

1. What are 3 or more ways to improve the effectiveness of the WA-MRH initiative in your view?

☐ Decision-making transparency e.g. publishing Public Assessment Reports

☐ Make publicly available any information that might help applicants in managing their submissions - templates of documents, lists of Q&A, timelines and milestones, disclosure of internal SOPs, etc.

☐ Consistency in application of guidelines and decisions

☐ Use of risk-based approaches e.g. reliance pathways

☐ Engagement and interaction with stakeholders

☐ Publishing of pending products

☐ Publishing of approved products

☐ Minimise the need for country specific documents

☐ Other (Please specify) ____

2. What are 3 or more ways to improve the efficiency of the WA-MRH initiative in your view?

☐ Specific and clear requirements made easily available to applicants

☐ Compliance with target timelines by measuring and monitoring each milestone in the review process

☐ Use of robust IT systems

☐ Transparency on metrics and statistics e.g. % completed within timeline

☐ Improved central tracking of WA-MRH products

☐ Improved resources e.g., number of assessors

☐ Centralised system for submission of applications and communication with applicants

☐ Other (please specify) _Expanding the Expert Committee's to include more resources available in the region________________________________

E: ENVISAGING THE STRATEGY FOR MOVING FORWARD

1. Rate the following proposals to improve the current WA-MRH operating model from 1 – 3, number 1 representing what you think would be **most effective** in improving efficiency and number 3 the **least effective**. *Enter the appropriate number in the space provided before each proposal.*

☐ To continue with the current operating model unchanged

☐ To continue with the current operating model and establish WA-MRH integrated information management system to manage and process applications.

☐ To continue with the current operating model but provide full information on the process including timelines and milestones as well as approved products on every participating country's website and on the WA-MRH website.

☐ The establishment of a regional administrative body to centrally receive and track WA-MRH applications which would be responsible for allocating work, apportioning the applicable fees to countries, tracking of applications and communication with applicants.

2. In your view, would the establishment of an ECOWAS regional medicines agency, if legally possible, be the best strategy for improved performance going forward? ☐ Yes ☐ No

   Please explain why?

3. In conclusion, what other strategies not previously highlighted can you think of that would strengthen the WA-MRH initiative going forward?

Please feel free to use the comment box below to elaborate on any of your answers or to highlight questions and answers that you believe should have been included in this questionnaire.

Name of person completing the questionnaire:

Title (position):
Date: ________________

Thank you for your time and help
